# ABCs of the degenerative spine

**DOI:** 10.1007/s13244-017-0584-z

**Published:** 2018-03-22

**Authors:** Sergiy V. Kushchayev, Tetiana Glushko, Mohamed Jarraya, Karl H. Schuleri, Mark C. Preul, Michael L. Brooks, Oleg M. Teytelboym

**Affiliations:** 1 Department of Radiology, Mercy Catholic Medical Center, 1500 Lansdowne Ave, Darby, PA 19023 USA; 2 Division of Neurological Surgery, Barrow Neurological Institute, St. Joseph’s Hospital and Medical Center, 350 West Thomas Rd, Phoenix, AZ USA

**Keywords:** Degenerative spine, Intervertebral disc herniation, Spondylosis, Modic changes, Spinal canal stenosis

## Abstract

**Abstract:**

Degenerative changes in the spine have high medical and socioeconomic significance. Imaging of the degenerative spine is a frequent challenge in radiology. The pathogenesis of this degenerative process represents a biomechanically related continuum of alterations, which can be identified with different imaging modalities. The aim of this article is to review radiological findings involving the intervertebral discs, end plates, bone marrow changes, facet joints and the spinal canal in relation to the pathogenesis of degenerative changes in the spine. Findings are described in association with the clinical symptoms they may cause, with a brief review of the possible treatment options. The article provides an illustrated review on the topic for radiology residents.

**Teaching Points:**

*• The adjacent vertebrae, intervertebral disc, ligaments and facet joints constitute a spinal unit.*

• *Degenerative change is a response to insults, such as mechanical or metabolic injury.*

• *Spine degeneration is a biomechanically related continuum of alterations evolving over time.*

## Introduction

**Fig. 1:**
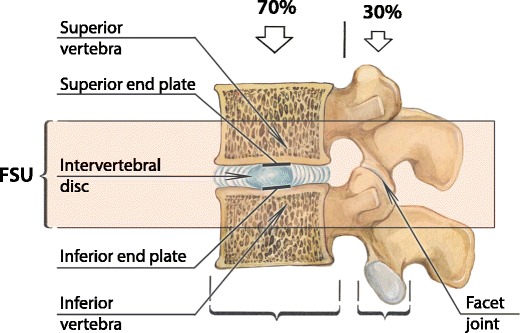
Functional spinal unit (FSU). The FSU represents the smallest motion segment of the spine and exhibits biomechanical characteristics similar to those of the entire spine. Approximately 70% of applied axial compression is transmitted by the vertebral body and the intervertebral discs, with the remaining 30% of the load being distributed through the facet joints

Erected vertically, the spine is the mast of our body and has three major functions: to provide structural support, enable trunk movement and protect the neural elements [1]. From a biomechanical point of view, the spine is a multiarticular structure comprising numerous segments or units, enabling multidirectional motions and the absorption of large complex loads. Two adjacent vertebrae, the intervertebral disc, spinal ligaments and facet joints between them constitute a *functional spinal unit* [2] (Fig. 1).

Degenerative change is considered a response to insults, such as mechanical or metabolic injury, rather than a disease [3]. The aetiology of the degenerative changes may be mechanical micro-insults or damage secondary to macro-insults, such as spinal fractures, spinal surgery not related to degenerative disc disease or significant metabolic processes, such as ochondrosis or mucoplysaccharidoses. All elements of the spine, including the intervertebral discs, joints, ligaments and bony structures, may undergo morphological changes that can be classified as degenerative.

Accurate and comprehensive interpretation of imaging findings relating to the degenerative spine can be challenging and sometimes even confusing because the word “degeneration” means different things to radiologists, neurologists, neurosurgeons and pathologists [3]. The pathogenesis of these changes in the spine is a biomechanically related continuum of alterations that evolve over time [4]. Therefore, understanding the pathophysiology of these biomechanical changes in the spine is essential for radiologists to characterise radiological abnormalities. The pathophysiology-based approach in assessing imaging findings in the degenerative spine can: (1) accurately characterise the process in the involved segment; (2) identify the sequence of degenerative changes and predict further abnormalities; (3) identify hidden or subtle abnormalities based on indirect signs; (4) assist clinicians in finding the source of pain or neurological symptoms; (5) identify the best treatment options for patients. No degenerative change should be considered an isolated event or reported as a random finding.

**Fig. 2:**
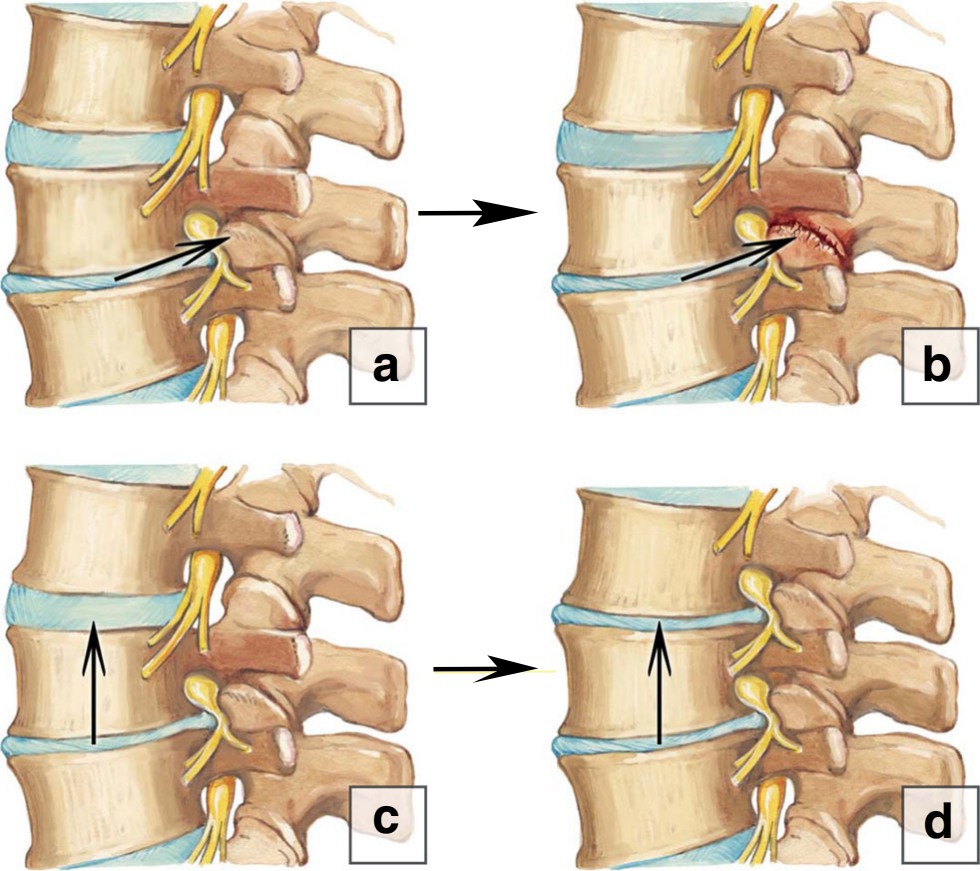
Types of spinal degeneration. (**a–b**) Horizontal degeneration. Initial degeneration of the intervertebral disc **(a)** subsequently leads to the facet joint osteoartritis **(b)**. (**c–d**) Adjacent segment disease. Severe degenerative changes on a segment result in abnormalities in the level above

**Fig. 3:**
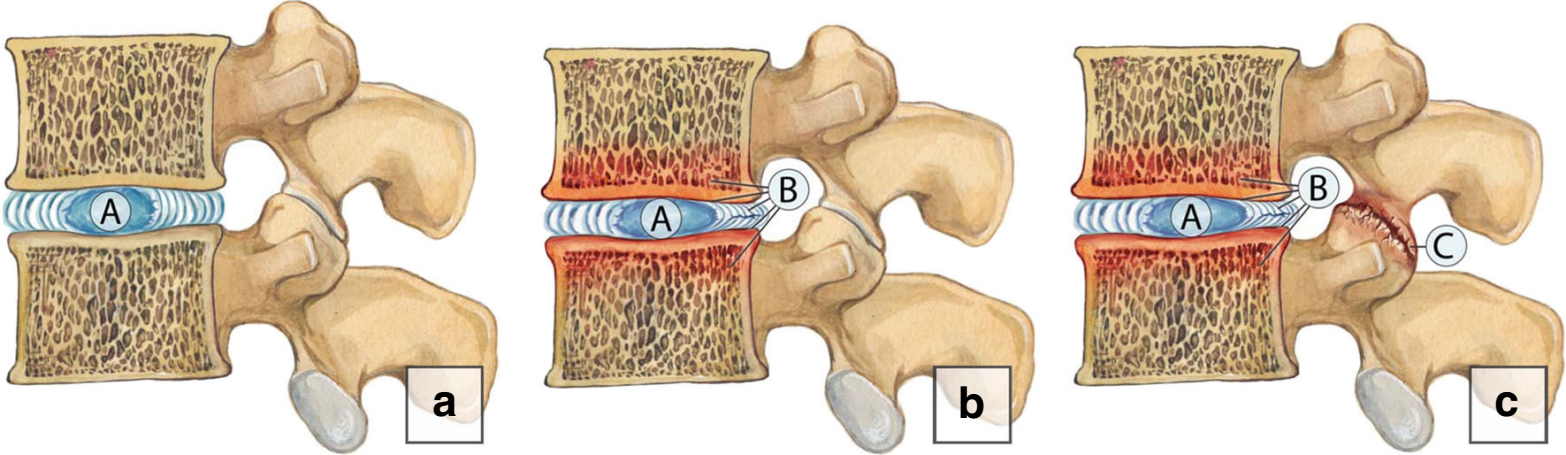
A-B-C degenerative changes. (**a**) A-changes. The degenerative process usually starts within the nucleous pulposus representing A-changes. (**b**) B-changes. The abnormalities extend to the disc, annulus fibrosus, end plates and bone marrow of the adjacent vertebral bodies. (**c**) C-changes. The advance degeneration may eventually involve distant structures and lead to facet joint osteoarthrosis, ligamentum flavum hypertrophy (not shown) and spinal canal stenosis (not shown)

Commonly, the degenerative process may include other elements of the involved functional spinal unit, which we term *horizontal or segmental degeneration* [5–7], or change the entire biomechanics of the spine, including the adjacent functional spinal units, know as an *adjacent segment disease* [8, 9] (Fig. 2). We propose a simple mnemonic and classification to facilitate description of spinal degenerative changes by dividing them into three categories of A, B and C changes, based on the location and sequence of progression. On imaging the degenerative process usually starts within the nucleolus pulposus (*A-changes*) and extend to the disc, annulus fibrosus, end plates and bone marrow of the adjacent vertebral bodies (*B-changes*). Advanced degeneration may eventually involve distant structures and lead to facet joint osteoarthritis, ligamentum flavum hypertrophy and spinal canal stenosis (***C-changes***) (Fig. 3).

## A-changes: nucleous pulposus

**Fig. 4:**
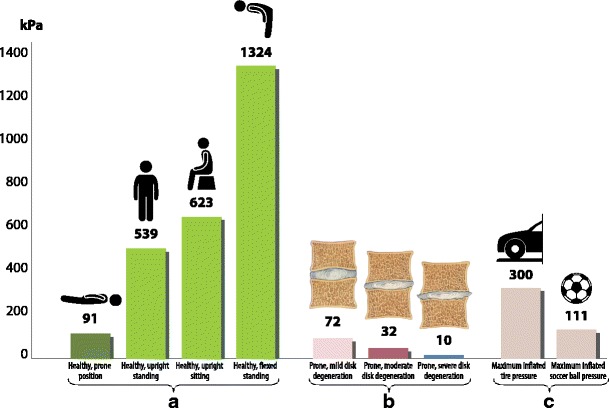
Intradiscal pressures. (**a**) The intradiscal pressures in the physiological postures in healthy individuals. (**b**) The intradiscal pressures in patients with mild, moderate and severe degeneration. (**c**) Maximum inflated pressures in tires and a soccer ball are presented for the purpose of comparison

In the majority of cases, the degenerative process starts with the nucleous pulposus. A normal nucleous pulposus is a gelatinous structure with high viscosity and elasticity, comprised of proteoglycans and intermolecular water (up to 80%) [10]. The chondrocytes provide a constant balanced turnover within the nucleous pulposus: they synthesise and break down the proteoglycans for the nucleous pulposus matrix that holds the water and collagen for the annulus fibrosus. A healthy intervertebral disc maintains a certain level of pressure, which is called the *intradiscal pressure* [10]. The mean intradiscal pressure on the L4–L5 discs in healthy individuals is about 91 kPa in the prone position, 151 kPa in the lateral position, 539 kPa in the upright standing position and 1324 kPa in the flexed standing position [10] (Fig. 4). A normal nucleous pulposus acts hydrostatically by transmitting evenly to the annulus fibrosus and end plates in every direction according to Pascal’s principle [10].

**Fig. 5:**
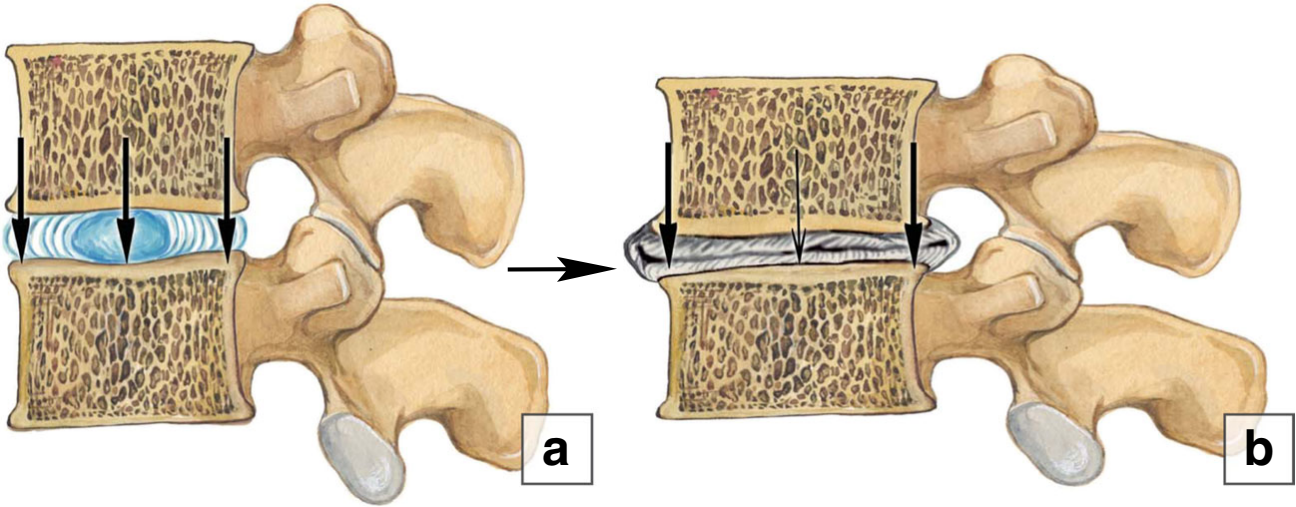
Stress distribution in a normal segment and in a segment with nucleous pulposus degeneration. (**a**) A schematic illustration of the normal balanced distribution of the loads in a disc. (**b**) In nucleus pulposus degeneration intradiscal pressure drops and the annulus fibrosus acts like a fibrous solid to resist compression directly

Abnormal mechanical axial stress owing to the combined effects of an unfavourable inheritance, age, inadequate metabolite transport and trauma impairs chondrocytes and can cause an nucleous pulposus to degenerate [11]. As degeneration progresses, the nucleous pulposus becomes desiccated resulting in reduced intradiscal pressure [10], thus passing the mechanical load on to the annulus fibrosus [1]. Because it has to hold greater weight, the annulus fibrosus undergoes changes to reflect the increasing strain it bears. Most of the annulus fibrosus then acts like a fibrous solid to resist compression (Fig. 5) [3, 11]. Increased stress on the annulus fibrosus can lead to development of cracks and cavities, subsequently progressing to clefts and fissures [12]. This loss of annulus fibrosus structural integrity may result in disc herniation. Structural weakness of the annulus fibrosus may also lead to the inability of the disc to maintain anatomical alignment and position progressing to instability and/or spondylolisthesis. All these structural changes are irreversible because adult discs have limited healing potential [11].

**Fig. 6:**
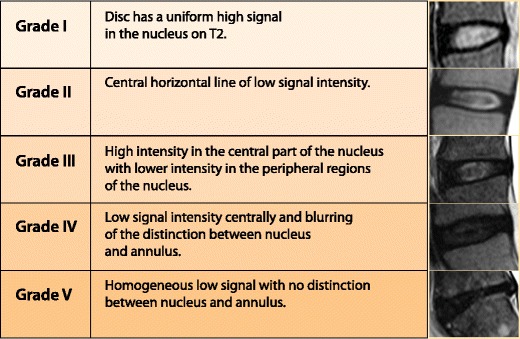
A grading system of intervertebral disk degeneration

On MRI, the hyperintense signal of the nucleus on T2-weighted images (WI) has been shown to correlate directly with the proteoglycan concentration in the nucleous pulposus and signal loss of the disc correlates with progressive degenerative changes [13, 14]. Pfirrmann et al. developed a grading system and algorithm based on MRI signal intensity, disc structure and distinctions among the nucleous pulposus, annulus fibrosus and disc height [14] (Fig. 6).

Novel functional imaging techniques, such as T2/T2* mapping, T1ρ calculation, T2 relaxation time measurement, diffusion quantitative imaging, chemical exchange saturation transfer, delayed contrast-enhanced MRI of cartilage, sodium-MRI and MR spectroscopy, are promising tools that allow the evaluation of early disc degeneration based on the chemical composition of a disc, mainly by evaluating the proteoglycan content [15]. These novel MRI techniques might be useful in the assessment of progression of disc degeneration and have potential applications in clinical trials to evaluate the efficacy of disc restoration therapies.

### Vacuum phenomenon

**Fig. 7:**
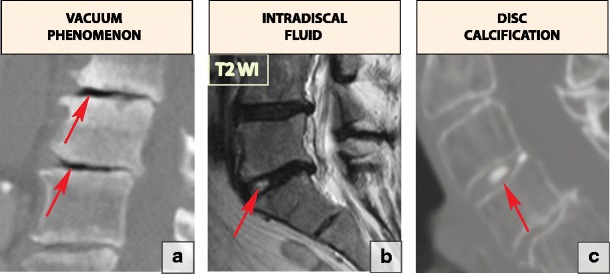
Signs of intervertebral disc degeneration: (**a**). The vacuum phenomenon. This sagittal CT reformatting image shows the foci of air within the L2–L3 and L3–L4 discs (arrows). (**b**) Intradiscal fluid accumulation (arrow). (**c**) A sagittal reformatting CT image at the level of C3–C4 shows disc calcification (arrow)

As disc degeneration progresses, nitrogen accumulates within the disc. This is a very rapid process and appears to be posture-dependent and often associated with segmental instability [16, 17]. On MRI, the vacuum phenomenon manifests as a signal void on both T1- and T2-WI [18] (Fig. 7a).

### Intradiscal fluid accumulation

Fluid in the disc is highly associated with the presence of the vacuum phenomenon, type 1 bone marrow changes (Modic 1) and severe end plate abnormalities. Fluid shows high signal on T2-WI and in the presence of type 1 Modic changes can mimic early spondylodiscitis [18] (Fig. 7b).

### Intradiscal calcification

Degenerative changes may lead to calcification of the disc. These changes most commonly involve the annulus fibrosus and are frequently located in the lower thoracic spine [19] (Fig. 7c).

## B-changes: annulus fibrosus, end plates and bone marrow

### Annular fissures

**Fig. 8:**
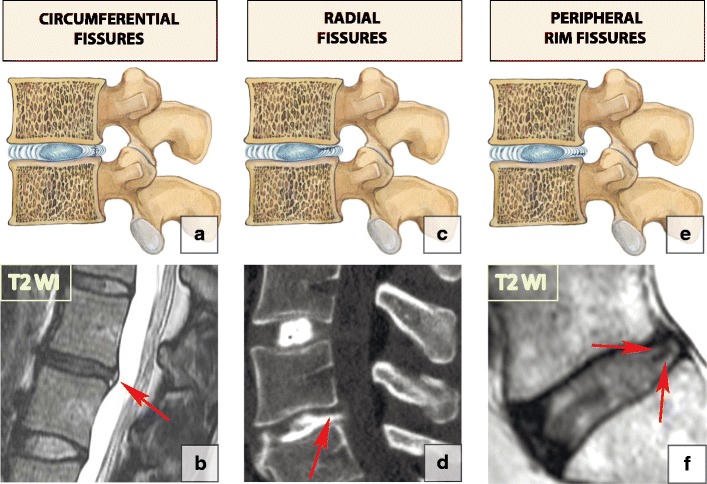
Annulus fibrous fissures: (**a–b**) Circumferential fissures. A drawing and an axial T2-WI scan at L4–L5 (arrow) showing a rupture of the transverse fibres without disruption of the longitudinal fibres representing circumferential fissures. (**c–d**) Radial fissures. A drawing and a sagittal CT discogram at L5–S1 showing (arrow) radial fissures extending from the periphery of the annulus to the nucleus, with disruption of the longitudinal fibres. (**e-f**) Peripheral rim fissures. A drawing and a sagittal T2-WI scan at L5–S1 demonstrating disruptions of Sharpey’s fibres at the annular periphery

Each annulus fibrosus comprises 15–20 collagenous laminae running obliquely from the edge of one vertebra down to the edge of the vertebra below and merging anteriorly and posteriorly with longitudinal ligaments. A normal outer annulus fibrosus shows a hypointense signal on all MRI sequences. The inner portion of the annulus fibrosus is made of fibrocartilage, which gradually blends with the nucleous pulposus; therefore, its MRI signal is similar to that of the nucleous pulposus. Annular tears or fissures are avulsions in the fibres of the annulus fibrosus and can either involve the fibres themselves or their insertions on the adjacent end plates [4]. A small amount of fluid tracking through the annulus fibrosus fissure is responsible for high-signal intensity on T2-WI; however, annulus fibrosus material is not displaced [11]. Annulus fibrosus fissures can be circumferential, peripheral rim and radial (Fig. 8). Since the fissures represent torn annulus fibrosus fibres and usually occur during excessive loading on the spine, acute fissures may clinically present with pain. The fissures do not change appearance on MRI over time and therefore cannot indicate the acuity of the process [20].

### Disc displacement

**Fig. 9:**
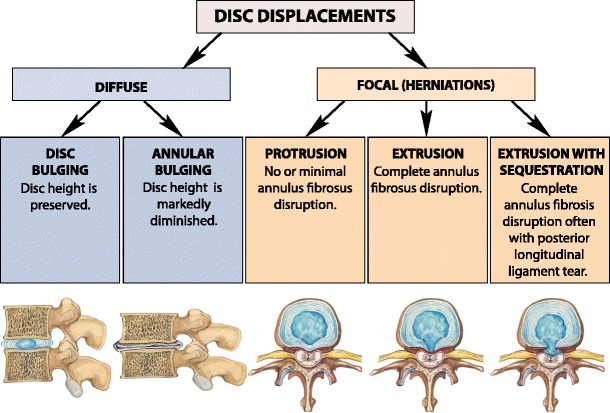
A classification of the disc displacements

**Fig. 10:**
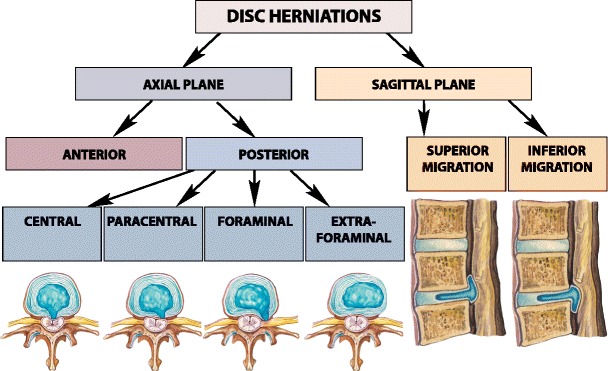
A classification of the focal disc displacements (herniations)

Displacement of disc material beyond the limits of the intervertebral disc space may be *diffuse* (bulging) or *focal herniation* (protrusion, extrusion and extrusion with sequestration) [21] (Fig. 9). On the axial plane, it may be anterior or posterior. Herniation can be classified as: *central*, *paracentral*, *foraminal* or *extraforaminal* [21–24]. The herniation may migrate *superiorly* or *inferiorly* [24] (Fig. 10).

Diffuse disc migration is the circumferential displacement of the annulus fibrosus.

**Fig. 11:**
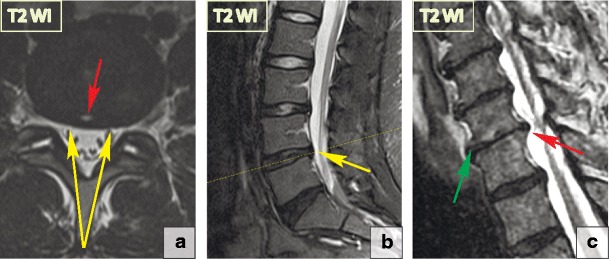
Diffuse displacement of the disc material: bulging and annular folding. (**a**) Disc bulging. There is a circumferential displacement of the L4–L5 disc (yellow arrows). Nevertheless, the height of the disc is preserved. Note that the focal hyperintensity within the posterior L4–L5 disc is compatible with the annulus fibrous fissure (red arrow). (**b**) Annular bulging at the C5–C6 level. The nucleous pulposus material has migrated anteriorly (green arrow), emptying the disc and resulting in severe disc space narrowing and the folding of the annulus fibrosus radially outward (red arrow)

- ***Disc bulging***. This occurs when intradiscal pressure remains high and the annulus fibrosus is intact and the height of the disc preserved. A rapid increase in intradiscal pressure in the setting of bulging may lead to the development of annular fissures and eventually result in herniation. Bulging is very often seen in asymptomatic individuals (Fig. 11a, b).
- ***Annular bulging*** (folding). Degeneration of the nucleous pulposus eventually leads to a marked drop in intradiscal pressure resulting in disc space narrowing or collapse with the vertebral bodies moving closer to one another. Increased vertical loading on the annulus fibrosus causes it to bulge or fold radially outward [25–27]. Annular bulging (folding) may be symptomatic as severe disc space narrowing also results in decreased size of the intervertebral foraminae, which is further exacerbated by bulging annulus fibrosus (Fig. 11c). Annular bulging (folding) has never been identified as a separate entity; however, it is an important finding from the clinical point of view, since the surgical treatment aims to restore the intervertebral disc space rather than microdiscectomy.

Focal disc migration (disc herniation) is defined as a condition where a detached piece of the nucleous pulposus migrates from its original intradiscal location. Herniation usually occurs in relatively young patients when intradiscal pressure remains high. Depending on the extent of the focal migration of the nucleous pulposus, disc herniation may result in protrusion, extrusion or sequestration of the nucleous pulposus material. Disc herniation may occur in any direction.

**Fig. 12:**
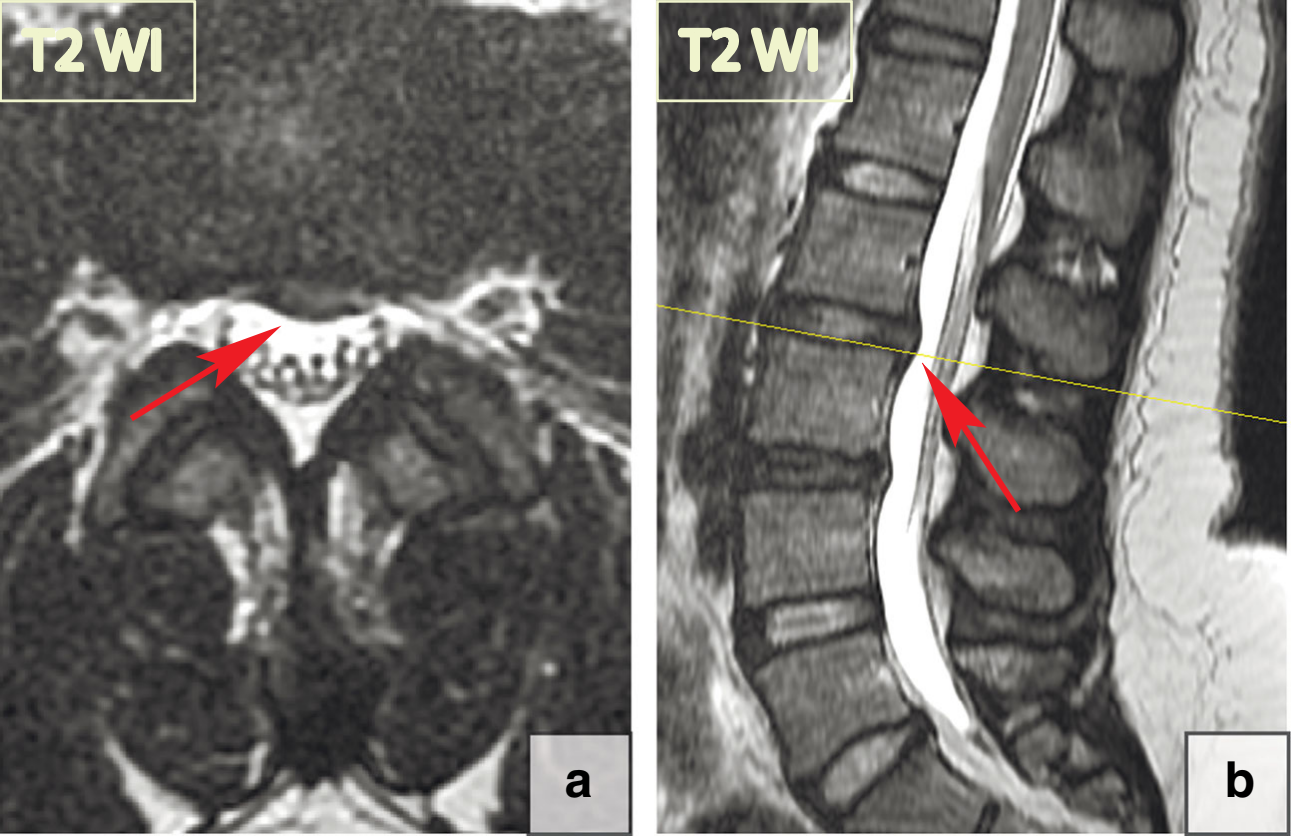
Focal disc displacement: protrusion. Axial and sagittal T2-WI scans demonstrate focal left L2-L3 paracentral posterior protrusion. There is no disruption of the fibres of the overlying annulus fibrosus or the posterior longitudinal ligament

**Fig. 13:**
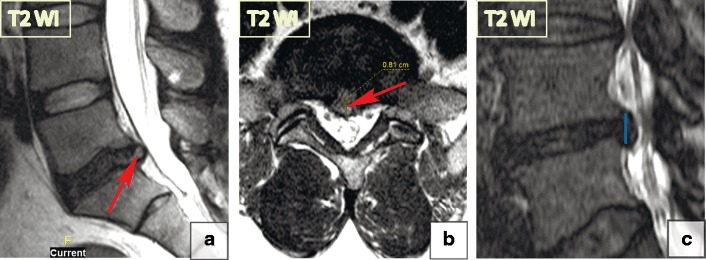
Focal disc displacement: extrusion. (**a–b**) There is an 8-mm focal central L5–S1 extrusion on the sagittal and axial T2-WI. (**c**) The image shows disc material displacement with complete disruption of the annulus fibrosus; however, the posterior longitudinal ligament remains intact. The posterior aspect of herniation (blue line) is larger than its base (red line) in the sagittal plane, consistent with a full thickness tear of the annulus fibrosus. The herniation material tents the posterior longitudinal ligament without tear. Thus, by definition, this abnormality is a disc extrusion

**Fig. 14:**
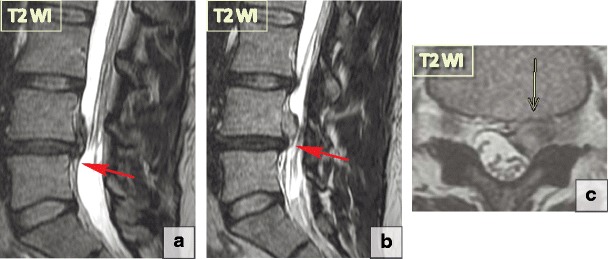
Focal disc displacement: extrusion with transligamentous sequestration. (**a–b**) Sagittal T2-WI scans demonstrate a large L4–L5 left-sided sequestered herniation with superior migration of the fragment. The disc material extends beyond the posterior longitudinal ligament margin suggesting its complete rupture. (**c**) The extruded disc material is round in the axial slides, which is a typical presentation

Consequently, based on their morphological appearance and imaging findings, herniations can be divided into three subtypes:

- ***Protrusion*** is described as localised (more than 25% of the circumference of the disc) displacement of disc material and the distance between the corresponding edges of the displaced portion must not be greater than the distance between the edges of the base of the displaced disc material at the disc space of origin [21]. Anatomically, protrusion is a focal displacement of disc material with no or minimal disruption of the fibres of the overlying annulus fibrosus and intact posterior longitudinal ligament (Fig. 12).
- ***Extrusion*** is a herniated disc in which, in at least one plane, any one distance between the edges of the disc material beyond the disc space is greater than the distance between the edges of the base of the disc material beyond the disc space in the same plane or when no continuity exists between the disc material beyond the disc space and that within the disc space [21]. Anatomically, the extrusion is the displacement of disc material with a full-thickness disruption of the annulus fibrosus fibres; usually the posterior longitudinal ligament however remains intact (Fig. 13). The posterior aspect of the extrusion may be larger than its base in the sagittal plane causing the posterior longitudinal ligament to tent, which often causes neurological symptoms and pain.
- ***Extrusion with sequestration*** is a focal disc displacement when extruded disc material that has no continuity with the disc of origin [21]. *A subligamentous sequestration* is a variant of an extrusion with sequestration, which occurs when the nucleous pulposus material splays along the posterior longitudinal ligament [21]. It appears spindle shaped on imaging. A *transligamentous sequestration* is when the disc material displacement results in full-thickness disruption of the annulus fibrosus fibres and posterior longitudinal ligament [21]. A fragment may stay at the level of the disc or may migrate superiorly or inferiorly. Pain and neurological symptoms may fluctuate with the migration of the free fragment within the spinal canal. The acute displacement of a free fragment from the disc into the spinal canal may cause acute cauda equina syndrome (Fig. 14).

Herniation directed posteriorly toward the spinal canal may have clinical significance as it can cause neuronal or spinal cord compression. However, annular fissures and acute disc herniation involving the anterior aspect of the disc can also be responsible for back pain. These are frequently are overlooked and underestimated.

There are no universally accepted radiological definitions of the intervals that distinguish among acute, subacute and chronic disc herniations [21]. From the neurological perspective, the patients with degenerative spines may present acute (lasting less than 4 weeks), subacute (lasting 4–12 weeks) and chronic (lasting more than 12 weeks) symptoms and pain [22, 28]. Acute disc herniations manifest with acute pain and neurological symptoms, subacute herniations correspond with subacute clinical presentations, and chronic herniations are accompanied by chronic symptoms and neurological signs.

**Fig. 15:**
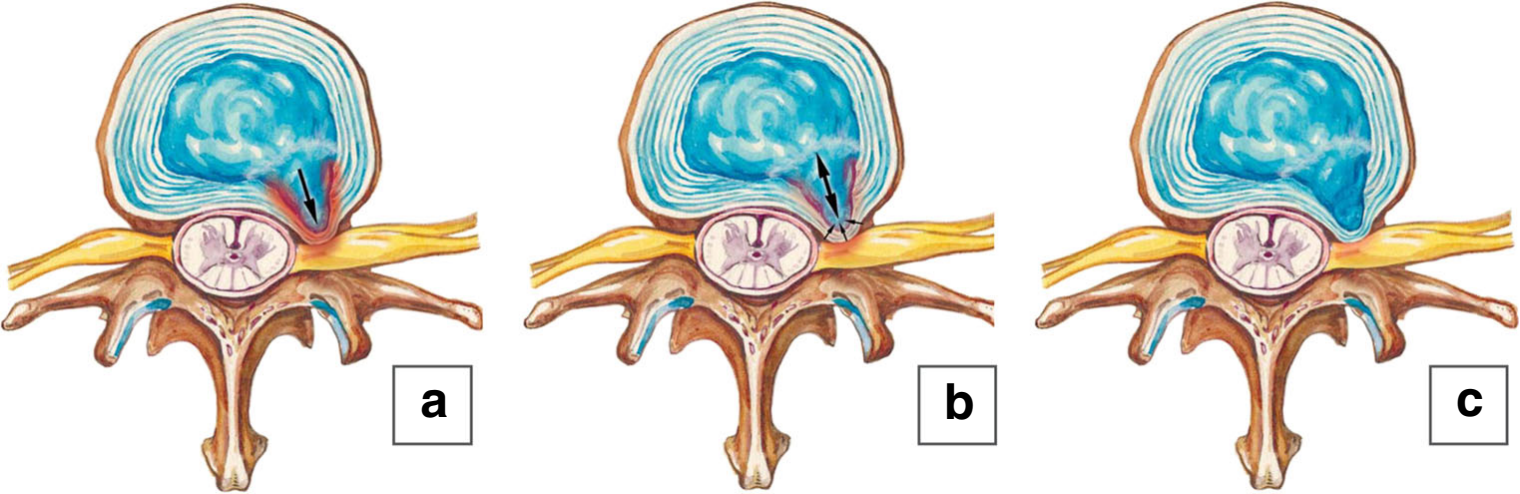
The stages of the nucleus pulposus displacement. The migrated intradiscal nucleous pulposus fragment displaces posteriorly. The arrows indicate the separation of the intradiscal fragment from the remaining nucleous pulposus material. (**a**) Acute herniation. It occurs at the early stages of degeneration when the intradiscal pressure is still relatively high. It causes the annulus fibrosus fibres to rupture and lead to acute local inflammation. (**b**) Subacute herniation. This usually arises only when the disc material migrates peripherally with increasing intradiscal pressure increases and improves when the intradiscal pressure drops. The remaining intact fibres of the annulus fibrosus recoil to bring the extruded material back into the disc space. (**a**) Chronic herniation. Chronic protrusions persist because of high intradiscal pressure pushing the nucleous pulposus material out of the disc

- *Acute herniations* occur in the early stage of degenerative disease when intradiscal pressure is still relatively high. Acute increases in intradiscal pressure in the setting of trauma or lifting heavy weights lead to the displacement of the nucleous pulposus through the compromised fibres of the annulus fibrosus, causing the annulus fibrosus fibres to rupture. Injured tissues show increased levels of catabolic cytokines and an acute focal inflammatory reaction [29]. Every episode of acute disc displacement leads to further migration of the nucleous pulposus posteriorly and worsening of the annulus fibrosus tear. Herniation without disc degeneration is rarely seen and typically occurs secondary to an acute traumatic event (Fig. 15a).
- *Subacute disc herniation* is associated with classically described back pain that worsens with standing and is better when the patient is lying down [30]. It arises only when disc material migrates peripherally as the intradiscal pressure increases (for example, in the standing position), but improves when intradiscal pressure drops (in the horizontal position) and the remaining intact fibres of the annulus fibrosus recoil to bring the extruded material back into the disc space. Since the majority of MRI and CT studies are performed in the prone position when the intradiscal pressure decreases, imaging findings may underestimate the extent of fluctuating nucleous pulposus displacement (Fig. 15b).
- *Chronic nucleous pulposus displacement* represents the stable displacement of the disc material outside the disc. In its early stage, chronic protrusions persist because of high intradiscal pressure pushing the nucleous pulposus material out of the disc; however, annulus fibrosus fibres later undergo advanced degenerative changes and lose the ability to recoil (Fig. 15c). Excessive axial stresses may lead to further migration of the intradiscal nucleous pulposus fragment, and additional tearing of the annulus fibrosus fibres results in the repetition of the acute stage. *Extrusion* occurs when the intradiscal fragment tears apart all the annulus fibrosus layers. Further migration of the extrusion leads to posterior longitudinal ligament tearing and *free disc fragments (sequester)* floating freely in the spinal canal.

Complications of disc displacement may be *neurological, vascular or focal. Neurological complications* are related to nerve root and spinal cord compression, which are the most common complications of disc herniation. At any spinal level, an acute persistent neurological deficit from disc herniation is a medical emergency, which may require surgical decompression. *Vascular complications* develop secondary to acute or chronic compression of the vertebral artery or medullary segmental arteries feeding the spinal cord (large cervical radiculomedullary at level C5–C7; dominant radiculomedullary artery at T4–T5; the artery of Adamkiewicz located at T10 and the additional radiculomedullary artery of Deproges-Gotteron arises at the L4–L5 level), which may cause a severe neurological deficit and also may require intervention. *Focal complications* occur because of long-standing inflammatory changes secondary to persistent fluctuating or chronic hernia, which eventually may lead to extensive epidural scarring (without surgical intervention). Normally, the nerve roots freely move in the foraminae with body movements. Epidural scarring limits nerve root passage through foraminae and may cause nerve root tethering. This process is virtually impossible to identify at imaging. Acute disc sequestration in the settings of adhesions between the ventral wall of the dura and the posterior longitudinal ligament may lead to dural perforation and developing intradural herniation. This is a very rare complication, comprising only 0.27% of all herniated discs and mostly occurring on the lumbar spine [31, 32]. Epidural vein varicosis, enlargement of epidural veins secondary to disc herniation usually on the lumbar spine, can mimic the clinical signs of disc herniation or spinal stenosis. MRI has been reported to be of high value in demonstrating the dilated epidural vein but the findings might be misinterpreted as herniated nucleus pulposus material [33].

Many studies have reported the spontaneous regression or disappearance of disc herniations without surgical management. Sequestrations have the highest likelihood of regressing radiographically in the shortest timeframe in comparison to the other subtypes of disc herniation. Although the exact mechanism of this phenomenon is unknown, dehydration and shrinkage appear to play a primary role and can be clearly demonstrated using MRI because of the decreasing water content over time [34]. After disc material sequestrates into the epidural space, it is recognised as a foreign body, and autoimmune and inflammatory responses lead to neovascularisation, enzymatic degradation and macrophage phagocytosis [34].

### End plate changes

**Fig. 16:**
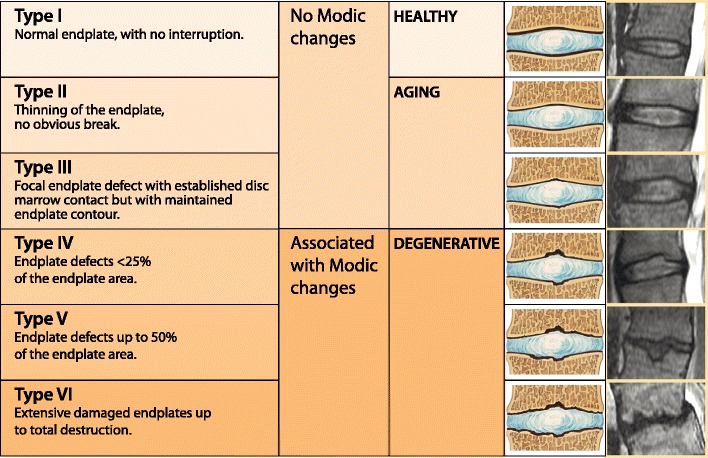
A classification of the end plate changes

End plates play a crucial role in the maintenance of the mechanical environment as well as the proper nutrition of avascular discs. End plate damage is the hallmark of degenerative changes. MRI-based end plate type classification is an objective method of differentiating healthy, ageing and degenerated discs. Six types of end plates have been identified according to the severity of the damage: type I is a normal end plate; type II indicates thin end plates without obvious breaks; type III denotes an end plate showing focal defects without subchondral bone changes; type IV signifies breaks involving less than 25% of the surface, usually associated with adjacent bone marrow changes; type V refers to large (up to 50%) end plate defects with associated bone marrow changes; type VI represents extensive end plate damage involving almost the entire end plate [35] (Fig. 16). End plate fractures lead to sudden depressurisation of the nucleous pulposus and the migration of the nucleous pulposus material into the vertebral body. This elicits an inflammatory response and oedema, which is detected on MRI as bone marrow (Modic) changes. Very large end plate damage with a large volume of migrated nucleous pulposus material usually indicates Schmorl’s nodules.

### Degenerative marrow changes

Although the exact causes of degenerative changes of the bone marrow (so-called Modic changes) are not clear, their occurrence may be closely related to mechanical stress [36]. The abnormal load and stress will affect vertebral end plates and the microenvironment of adjacent vertebral bone marrow, resulting in histological changes, which exhibit signal intensity change on MRI [36]. There are three main forms of degenerative change involving the bone marrow of the adjacent vertebral bodies. These may also occur in the pedicles [37].

**Fig. 17:**
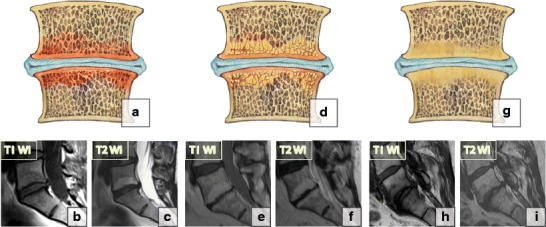
Degenerative bone marrow (Modic) changes. (**a–c**) Type 1 changes. (**d–f**) Type 2 changes. (**g–i**) Type 3 changes

Type 1 changes (decreased signal intensity on T1-WI and increased signal intensity on T2-WI, enhancement after contrast administration) correspond to *bone marrow oedema* and *vascularised fibrous tissues (*Fig. 17a–c). These changes are found in 4% of patients scanned for lumbar disease, in up to 30% of patients after discectomy and in 40–50% of chymopapain-treated discs [38, 39]. Type 1 changes may be chronic or acute and are strongly associated with non-specific lumbar pain [38] and instability. The aetiology of Modic 1 changes remains unclear; they appear to have biomechanical and biochemical causes. The proposed biomechanical mechanism involves fissuring and microfractures of the end plate due to the uneven distribution of loads across the disc resulting from disc degeneration. After cumulative trauma, this leads to oedema and vascularisation. Biochemically, intravertebral migration of the nucleous pulposus material with a high concentration of inflammatory substances secondary to trauma and degeneration results in a local bone marrow [18] inflammatory reaction, which in turn gives rise to back pain. Type 1 changes have been noted to slowly convert to type 2; however, reverse reconversion has also been reported [40].

**Fig. 18:**
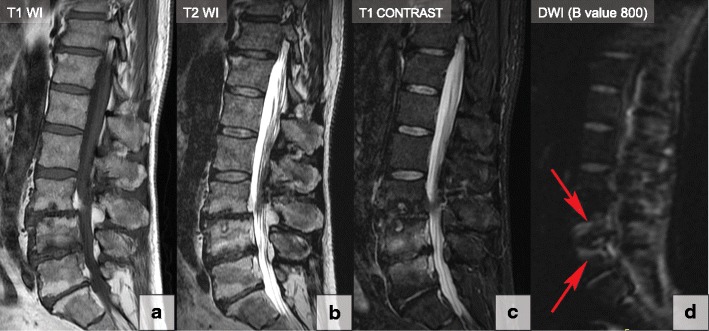
The claw sign in type 1 degenerative bone marrow (Modic) changes at L4–L5. T1-WI (**a**), T2-WI (**b**), T1-contrast-enhanced (**c**) and DWI sagittal images (B value 800) (**d**). The claw sign is identified in the DWI image as linear paired regions of high signal located within adjusted vertebral bodies at the boundaries between the normal and vascularised bone marrow (red arrows). Please note that type 2 degenerative bone marrow changes at L5–S1 and L3–L4 do not demonstrate the claw sign

Modic type 1 degenerative signal changes may mimic or suggest infection. Diffusion-weighted imaging (DWI) is useful for differentiating degenerative and infectious end plate abnormalities. Modic type 1 changes show the claw sign on DWI when presenting as well-marginated, linear, typically paired regions of high signal situated within the adjoining vertebral bodies at the boundaries between the normal bone marrow and vascularised bone marrow that lies close to the affected disc (Fig. 18). Slow progressive degenerative disc disease produces a well-defined border response. Conversely, the infection process may progress very quickly, becoming diffusely infiltrated with pathogens or oedema, and thus fail to produce a defined border zone response so that the claw sign is absent [41, 42].

Type 2 changes (increased on T1-WI and iso/hyperintense on T2-WI without contrast enhancement) reflect the presence of *yellow marrow* in the vertebral bodies (Fig. 17d–f).

Type 3 changes (decreased on both T1- and T2-WI) represent *dense woven bone* and the absence of marrow. These changes are potentially stable and almost always asymptomatic (Fig. 17g–i).

### Degenerative intervertebral instability

**Fig. 19:**
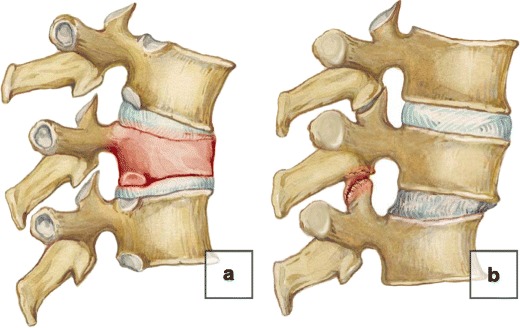
Vertical and horizontal instability of the spine. (**a**) Vertical instability in the settings of a vertebral body fracture. (**b**) Horizontal instability in spondylolystesis

Biomechanically, the spinal stability is considered in both the vertical axis and transverse plane. Axial (vertical) instability of the spine is usually related to processes involving vertebral bodies can be due to *focal* (traumatic fracture or large lytic lesion) or *diffuse* (such as osteoporosis or multiple myeloma) pathology [43, 44] (Fig. 19a). Horizontal (intervertebral or segmental instability) is the inability of the intervertebral disc, facet joints and ligamentous apparatus to maintain the anatomical alignment and anatomical position of the involved functional spinal unit [17]. It may occur in degenerative spondylolysis, spondylodiscitis and other processes (Fig. 19b). Degenerative instability may occur in the cervical or lumbar spine and almost never occurs in the thoracic spine. The process of degenerative instability is divided into three phases: early dysfunction, instability and stabilisation [29]. Degenerative instability consists of *pure motion dysfunctional syndrome* with no or minimal anatomical changes (*microinstability*), undetectable on imaging, and *overt instability*, which can be detected radiologically [45]. Differentiation between normal and abnormal motion remains challenging, and a diagnosis of intervertebral instability is based on both the direct and indirect radiological findings of abnormal vertebral motion. Persistent uni- or multisegmental instability produces rotational and translational subluxation, resulting in degenerative spondylolisthesis [46].

Clinically, instability presents with intermittent nonspecific back pain that worsens with movement. A variety of imaging modalities are currently used to assess spinal instability. Conventional MRI and CT performed in the prone position provide limited information on the functional status of the affected segment as spondylolisthesis with instability may “self-reduce” without a normal axial load. These techniques can demonstrate indirect signs of instability, such as the presence of traction spurs, intradiscal vacuum phenomenon or ligamentum flavum hypertrophy. Functional modalities, such as kinetic MRI and flexion and extension radiographs, are effective ways to evaluate abnormal motions in the involved segment. For the lumbar spine, on flexion-extension radiographs values of 10° for sagittal rotation and 4 mm for sagittal translation are typically used to infer instability [17]. Specific criteria for the diagnosis of instability of the cervical spine have not yet been established: transitions from 1 mm to 3.5 mm on functional radiographs have been proposed in the literature [47], and a 3-mm slippage appears to be a reliable cut-off. The CT twist test is obtained through the facet joint while the patient twists his/her body and the pelvis is tightly strapped to the CT table. The clinical significance of the twist test is not well established.

### Degenerative spondylolisthesis

**Fig. 20:**
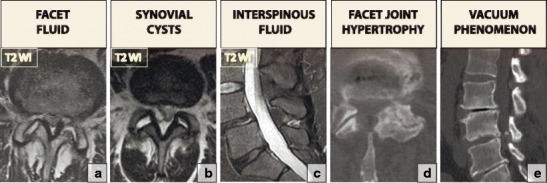
Features that are suggestive of the presence of instability in spondylolisthesis: (**a**) facet fluid, (**b**) synovial cyst, (**c**) interspinous fluid, (**d**) facet joint hypertrophy and (**e**) the vacuum phenomenon

Degenerative spondylolisthesis is most commonly seen in the lumbar spine and virtually never occurs in the thoracic spine. Cervical spondylolisthesis has not been extensively studied but may be more common than previously thought [47]. The mechanisms for the formation of spondylolisthesis seem to be similar throughout the spine; the condition represents the result of severe disc degeneration. Degenerative spondylolisthesis is divided into *dynamic spondylolisthesis*, which demonstrates instability on flexion/extension radiographs, and the *static* subtype, which does not show radiological evidence of instability [48]. The presence of indirect signs of instability, namely facet fluid, facet synovial cysts, interspinous fluid, facet hypertrophy and the intradiscal vacuum phenomenon on MRI, is suggestive of instability (Fig. 20). Functional flexion/extension radiographs are considered the gold standard for diagnosing the presence of degenerative instability in the setting of spondylolisthesis [48]. The static spondylolisthesis subtype may not necessarily need instrumentation or fusion, whereas dynamic subtypes may require additional fixation.

#### Cervical spondylolisthesis

**Fig. 21:**
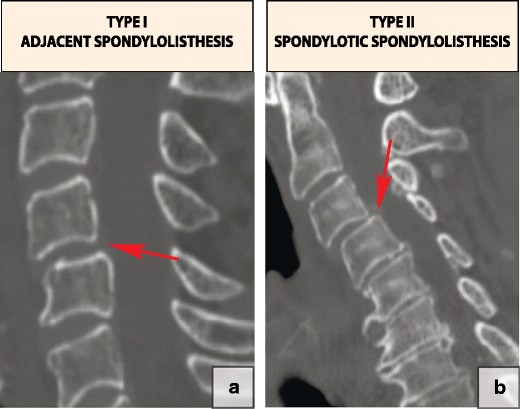
A classification of cervical degenerative spondylolisthesis: (**a**) type I, adjacent spondylolisthesis (arrow); (**b**) type II, spondylotic spondylolisthesis (arrow)

Two radiographically distinct types of cervical degenerative spondylolisthesis have been described: type I, *adjacent spondylolisthesis*, which occurs adjacent to a relatively stiffer spondylotic segment at the transition from stiff to more mobile segments, and type II, *spondylotic spondylolisthesis*, which develops within spondylotic cervical segments and is associated with advanced disc degeneration (Fig. 21) [49].

#### Lumbar spondylolisthesis

**Fig. 22:**
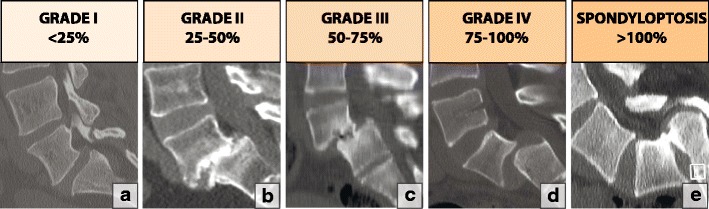
A classification of lumbar degenerative spondylolisthesis

A commonly used method of grading spondylolisthesis is the Meyerding classification, which is based on the ratio of the overhanging part of the superior vertebral body to the anteroposterior length of the adjacent inferior vertebral body (Fig. 22).

### Spondylosis

**Fig. 23:**
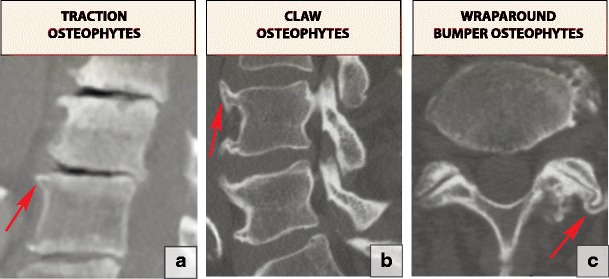
Three types of osteophytes related to the degenerative spine: (**a**) traction osteophytes (arrow), (**b**) claw osteophytes (arrow) and (**c**) wraparound bumper osteophytes (arrow)

Spondylosis is common nonspecific term used to describe hypertrophic changes of the end plates (osteophytes) and facet joints. There are three types of true degenerative osteophytes: traction osteophytes (Fig. 23a) are 2–3-mm bony structures projecting in a horizontal direction, while claw osteophytes (Fig. 23b) have a sweeping configuration toward the corresponding part of the vertebral body opposite the disc. Traction and claw osteophytes frequently co-exist on the same vertebral rim and are associated with horizontal instability. They result from increased flexibility between the vertebral bodies and the production of inhomogeneous mechanical stress on the annulus fibrosus and edges of the vertebral body, with subsequent sclerotic or hyperplastic changes occurring on the edges of the vertebral bodies [50, 51]. A wraparound bumper (Fig. 23c) develops along the capsular insertion of the facet joints and is believed to be associated with instability [45].

### Treatment

**Fig. 24:**
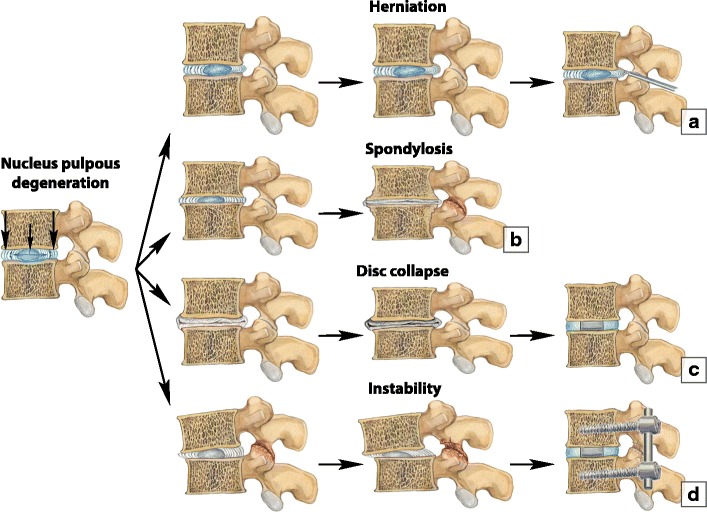
Surgical treatment options for degenerative changes. (**a**) Herniations. Herniations are often associated with pain and neurological symptoms and most commonly occur on the lumbar spine. Treatment options include conservative treatment if the herniation does not compress the nerves and surgical removal of the herniation if neural compression exists. (**b**) Spondylosis. Chronic longstanding disc degeneration results in slowly progressive mild-to-moderate disc space narrowing and gradual osteophyte formation without apparent disc displacement. Spondylosis is considered an adaptive reaction to stabilise motion in the presence of instability or a compensatory mechanism to limit the range of motion and prevent further degeneration. Altered disc biomechanics and narrowed intervertebral disc space subsequently lead to facet joint degeneration. This is probably the most favourable type of degeneration as it is essentially asymptomatic, may be seen at all levels of the spine and typically does not require treatment. (**c**) Disc collapse. Disc collapse leads to annular folding, anterior bulging of the flaval ligaments and posterior bulging of the posterior longitudinal ligament, with consequential narrowing of the central spinal canal. The decreasing disc height of the involved spinal segment leads to increased stiffness and may cause vertical degeneration of the adjacent vertebral segments. (**d**) Progressive structural failure of the disc to maintain the integrity of the functional spinal unit leads to segmental instability. This may progress to degenerative spondylolysthesis and require spinal instrumentation

Conservative treatment is considered a first-line treatment for the majority of patients with degenerative spine disease, unless the disease presents with acute neurological symptoms such as myelopathy or cauda equine syndrome. When medical therapy fails, imaging and spinal surgery are considered as the next step of management. Depending on the prevalent degenerative pattern, different surgical approaches can be utilised. Patients with symptomatic disc herniations can benefit from microdiscectomy [52]. If a degenerative process results in destabilisation and abnormal spinal motion, different types of surgical fusion can be used to stabilise the spine [53]. Interbody fusion implants are widely used to restore disc height and support the anterior column [54]. Spondylotic changes usually do not require surgery (Fig. 24).

## C-changes: facet joints, figamentum flavum and spinal canal

### Degenerative changes in the facet joints

**Fig. 25:**
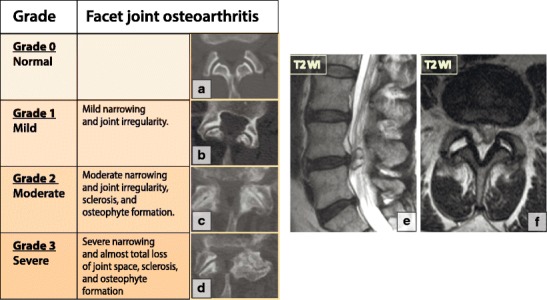
Degenerative changes of the facet joints. (**a–d**) Radiological classification of facet joint osteoarthritis. (**e–f**) A synovial cyst at L4–L5. Bilateral degenerated facet joint effusions with a left-sided synovial cyst compressing the left dorsal aspect of the thecal sac

The facet joints, which are true synovial joints, are present at every spinal level except C1–C2. Although facet joint osteoarthritis may occur independently and be a source of symptoms on its own, it typically represents a secondary process that is associated with disc degeneration and loss of disc space height. Facet joint osteoarthritis leads to increased stresses on the facet joints and results in craniocaudal subluxation, arthrosis and osteophytosis [13]. A four-tiered grading scale has been proposed to assess facet joint osteoarthritis [55] (Fig. 25). Hypertrophic facet joint osteoarthritis (OA) can result in narrowing of the central canal, lateral recesses and foramina [39]. Several types of symptoms may be associated with facet joint osteoarthritis. Treatment of all types of spine joint osteoarthritis is conservative unless hypertrophic changes cause compression of the neuronal structures or spinal cord.

Bulging of the synovium through the facet joint capsule, especially in the presence of instability, may result in synovial cysts [13]. The majority (about 90%) of synovial cysts are found at the L4–L5 level and present clinically with lumbar radiculopathy. On MRI, synovial cysts are hyperintense on T2-WI if there is direct communication with the facet joint and hyperintense on T1-WI if there is a haemorrhagic or proteinaceous component. If clinically significant, a synovial cyst may require percutaneous fenestration or open surgery (Fig. 25).

### Ligamentum flavum hypertrophy

**Fig. 26:**
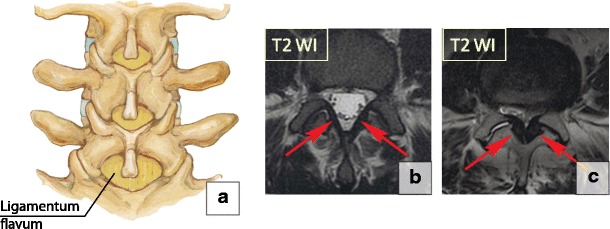
Ligamentum flavum. (**a**) A drawing of the normal anatomy of the ligamentum flavum. (**b**) Normal ligamentum flavum (arrows) on axial T2-WI scans. (**c**) Severe hypertrophy of the ligamentum flavum on sagittal T2-WI (arrows). Note that there is fluid in the right facet joint, suggestive of segmental instability

The ligamentum flavum, called the yellow ligament because of the high content of yellow elastin, makes up about 60–70% of the extracellular matrix. It extends from the second cervical vertebra to the first sacral vertebra, thus connecting the two adjacent laminae (Fig. 26a). The ligamentum flavum tends to become hypertrophic with the degeneration of the elastic fibres and the proliferation of type II collagen. Thickening of the ligamentum flavum is correlated with disc degeneration and herniation [56]. Abnormal motions and instability within the involved segments are potential aetiologies of ligamentum flavum hypertrophy as the body tries to stabilise the diseased segment by making it harder and thicker [57, 58]. Ligamentum flavum hypertrophy reduces the diameter of the spinal canal posteriorly and is considered an important causative factor in the development of lumbar spinal stenosis. Surgical removal is the only therapeutic manoeuvre for patients with symptoms caused by ligamentum flavum hypertrophy (Fig. 26b, c).

### Spinal canal stenosis

**Fig. 27:**
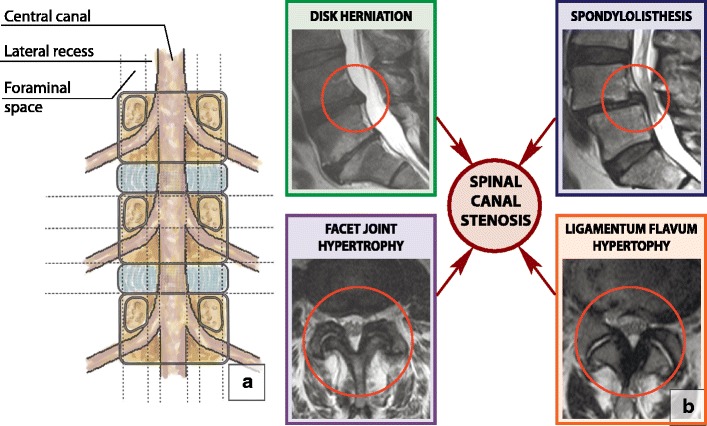
Spinal canal. (**a**) Normal spinal canal. The central portion of the spinal canal is bordered laterally by a lateral recess, dorsally by a vertebral arch and ventrally by a vertebral body and discs. The lateral recess is bordered laterally by a pedicle, dorsally by a superior articular facet and ventrally by a vertebral body and discs. The foraminal space is bordered by cephalad and caudal pedicles and facet joints dorsally and a vertebral body and discs ventrally. The extraforaminal space is lateral to the neuroforamen. (**b**) Spinal canal stenosis. There are four major causes of degenerative spinal canal stenosis: disc herniation, hypertrophic facet joint osteoarthrosis, ligamentum flavum hypertrophy and degenerative spondylolisthesis

Spinal canal stenosis refers to the diverse conditions that decrease the total area of the spinal canal, lateral recesses or neural foramina [59] (Fig. 27a). It is generally divided into developmental or congenital and acquired types. Four factors are associated with the degenerative changes of the spine that cause spinal canal stenosis: disc herniation, hypertrophic facet joint osteoarthritis, ligamentum flavum hypertrophy and spondylolisthesis (Fig. 27b). Anatomically, a spinal canal with stenosis can be divided into central, lateral and foraminal. Stenosis can occur in each of these parts, which should be assessed separately. Grading systems based on spinal canal measurements appear impractical; therefore, qualitative assessment of the relationships between the anatomical structures plays a major role in establishing the presence of spinal canal stenosis. Kang’s system for cervical spinal canal stenosis [60], Park’s classification for foraminal cervical and central lumbar stenosis [61, 62], Bartynski’s grading system for lumbar central stenosis [63] and Wildermuth’s categorisation of foraminal stenosis [64] can be easily used in clinical practice. The proposed systems are consistent and straightforward: grade 0 means no stenosis, grade 1 is mild stenosis, grade 2 refers to moderate stenosis and grade 3 indicates severe stenosis [63, 64].

#### Cervical spinal canal stenosis

**Fig. 28:**
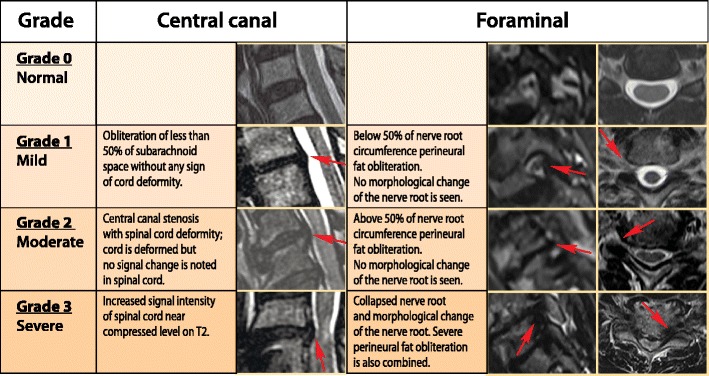
A grading system of cervical central and foraminal stenosis on the cervical spine

An MRI grading system for cervical central canal stenosis ranks stenosis in grades: grade 0 (no stenosis), grade 1 (obliteration of less than 50% of the subarachnoid space without any sign of cord deformity), grade 2 (central canal stenosis with spinal cord deformity; the cord is deformed but no signal change is noted in the spinal cord) and grade 3 (stenosis with increased signal intensity of the spinal cord reflecting myelomalacia) (Fig. 28) [60]. The severity of foraminal stenosis in the cervical spine can be assessed using a three-tiered grading system: grade 0 refers to the absence of foraminal stenosis; grade 1 denotes mild foraminal stenosis showing partial (less than 50% of the root circumference) perineural fat obliteration surrounding the nerve root without evidence of morphological changes in the nerve root; grade 2 is moderate (less than 50% of the root circumference) foraminal stenosis with nearly complete perineural fat obliteration surrounding the nerve root without morphological changes in the nerve root; grade 3 indicates severe foraminal stenosis showing nerve root collapse [61] (Fig. 28).

#### Lumbar spinal canal stenosis

**Fig. 29:**
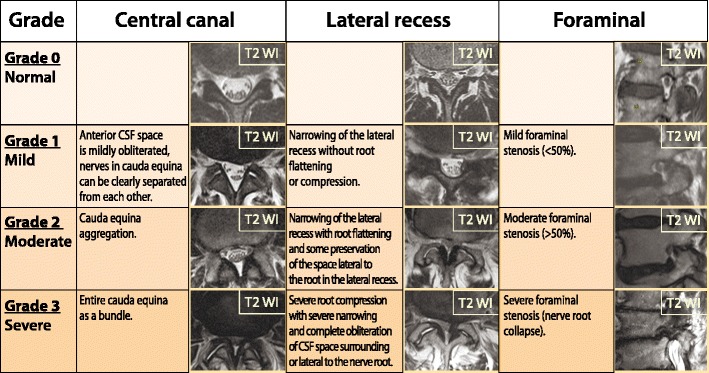
A grading of severity of central, lateral and foraminal stenosis on the lumbar spine

Central spinal canal stenosis of the lumbar spine can be classified based on the cauda equina nerve root aggregation. Grade 1 (mild stenosis) is when the anterior CSF space is mildly obliterated, but all the nerves in the cauda equina can be clearly separated from each other. Grade 2 or moderate stenosis indicates cauda equina aggregation, while grade 3 signifies severe stenosis with the entire cauda equina appearing as a bundle (Fig. 29) [62].

Lumbar lateral canal stenosis can be classified as: grade 0, no stenosis; grade 1, mild stenosis, where there is narrowing of the lateral recess without root flattening or compression; grade 2, moderate stenosis, where further narrowing of the lateral recess occurs with root flattening but there is some preservation of the space lateral to the root in the lateral recess; grade 3, severe stenosis in which there is severe root compression with severe narrowing and complete obliteration of the CSF space surrounding or lateral to the nerve root (Fig. 29) [63].

Lumbar foraminal stenosis can be absent (grade 0), mild (grade 1, with deformity of the epidural fat while the remaining fat still completely surrounds the existing nerve root), moderate (grade 2, with marked foraminal stenosis where epidural fat only partially surrounds the nerve root) and severe (grade 3 or advanced stenosis, with complete obliteration of the foraminal epidural fat) (Fig. 29) [64].

## Other findings of the degenerative spine

### Atlanto-occipital joint

**Fig. 30:**
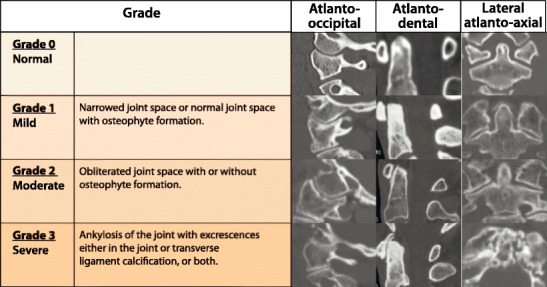
Grading of severity of degenerative changes in the atlanto-occipital, atlanto-dental and lateral atlanto-axial joints

The presenting symptoms of osteoarthritis of the atlanto-occipital joint are similar to the symptoms in the atlanto-axial segment; patients have unremitting unilateral suboccipital pain [65] (Fig. 30).

### Lateral atlanto-axial joints

The incidence of lateral atlanto-axial osteoarthritis in the elderly population varies from 4% to 18% [66]. Patients with atlanto-axial arthritis may suffer from suboccipital pain that is exacerbated by head rotation and distinct from other types of cervicalgia and headaches. The severity of the osteoarthritis is graded as none, mild, moderate and severe in each joint [67] (Fig. 30).

### Atlanto-odontoid joint

The atlanto-odontoid joint contributes between 40% and 70% to total cervical spine rotation [68]. The incidence of degenerative changes in this joint in the normal population is quite high, with 42% in the 7th decade and 61% in the 8th decade [69]. The severity of the osteoarthritis can be graded as none, mild, moderate and severe [67]. Rarely atlanto-dental OA complicated with hypertrophic changes is the cause of cervical myelopathy [70] (Fig. 30).

### Uncovertebral joints

**Fig. 31:**
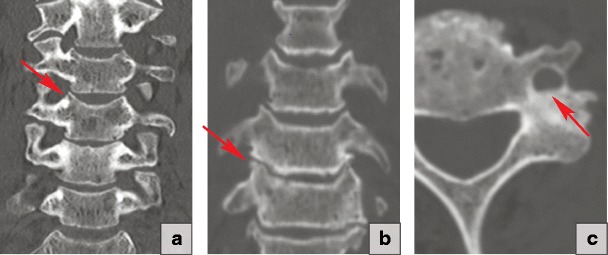
Uncovertebral joint. (**a**) Normal uncovertebral joint. The joint (arrow) is formed by uncinate processes above and below. (**b**) Uncovertebral arthrosis. Degenerative changes involving uncovertebral joints lead to uncovertebral arthrosis (arrow). (**c**) Hypertrophic uncovertebral arthrosis. Hypertrophic degenerative changes in the uncovertebral joint may result in foramen transversarium narrowing (arrow) and even vertebral artery compromise

The uncinate process and the associated uncovertebral articulation are also important in providing stability and guiding the motion of the cervical spine with degeneration; they become clinically apparent with compression of the adjacent nerve root and vertebral artery [71, 72] (Fig. 31).

### Diffuse idiopathic skeletal hyperostosis (DISH) of the spine or Forestier disease

The condition is characterised by continuous ossification of ligaments and enthuses of the spine. The coarse and thick bony spinal bridges form along the anterior longitudinal ligament in a more horizontal orientation and mainly on the right side [73]. The commonly accepted Resnick and Niwayama classification criteria for the spine require flowing osteophytes over four vertebral bodies and in addition the preservation of the intervertebral disc space [74]. Ankylosis of the spine in patients with DISH increases the risk of spinal fracture four-fold; fractures may occur even after relatively low-energy trauma and often display highly unstable fracture configurations [75].

## DTI/tractography and the degenerative spine

Diffusion tensor imaging with fibre tracking has found clinical applications in the evaluation of the compressed spinal cord and nerve roots and visualisation of abnormal nerve tracts [76, 77].

## Senescence of the spine

Senescence of the spine is a normal part of ageing [78]. It resembles changes in other ageing collagenous tissues and is unassociated with pain [11]. With age the intervertebral discs become drier, more fibrous and stiffer secondary to decreased water-binding power, which makes them less able to recover from deformation. Nevertheless, the boundary between physiological disc ageing and early degenerative changes is not always clear since in most cases ageing and degenerative changes do not substantially differ on imaging. The imaging findings that are not associated with senescence and should be considered as degenerative include annular fissures, disc herniations, end plate changes, degenerative bone marrow changes, instability/spondylolisthesis and spinal canal stenosis.

## Metabolic causes of degenerative changes

**Fig. 32:**
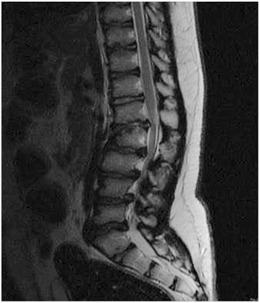
Secondary degenerative changes of the spine. Sagittal T2-WI of a 17-year old male with Hunter syndrome. Multilevel nulceous pulposus degeneration, advanced end plate changes and focal disc displacements are present. The L2 vertebral body shows anterior breaking, resulting in mild kyphosis

Mucopolysaccharidoses (Hunter syndrome, Sanfilippo syndrome, Morquio syndrome), diabetes mellitus and ochronosis are considered specific causes of degenerative changes [79, 80]. Mucoplysaccharidoses have a direct impact on cartilage and bone development resulting in advance degenerative changes in the spine (Fig. 32). The intervertebral discs of patients with diabetes mellitus have decreased hexosamine content, deficiencies in proteoglycan synthesis and reduced concentrations of keratosulphate, which is a critical component of proteoglycans [3]. Ochronosis produces deposits of a black pigment derived from homogentisic acid, which ostensibly impedes the normal metabolism of the disc matrix [3].

## Clinical aspects of degenerative spine disease and reporting

The role of imaging is to provide accurate morphological information and influence therapeutic decision-making [39]. The presence of degenerative change is not itself an indicator of symptoms. In the majority of cases, when patients with degenerative spine diseases are referred for imaging, clinicians are looking for answers to two simple questions: what is the cause of the patient’s pain or neurological symptoms, and what treatment option should be primarily considered in this particular situation? Therefore, these imaging findings must be interpreted in the context of the patient’s clinical condition. In the majority of cases, even in advanced cases of degenerative spine disease with multilevel involvement, it is possible to identify one leading cause of the patient’s problem or to provide a list of potential choices or culprits so that the referring physician can select the right answer based on the presentation, clinical symptoms and physical examination of the patient.
